# Host-ant specificity of endangered large blue butterflies (*Phengaris* spp., Lepidoptera: Lycaenidae) in Japan

**DOI:** 10.1038/srep36364

**Published:** 2016-11-03

**Authors:** Shouhei Ueda, Takashi Komatsu, Takao Itino, Ryusuke Arai, Hironori Sakamoto

**Affiliations:** 1 Department of Biology, Faculty of Science, Shinshu University, Asahi 3-1-1, Matsumoto, Nagano 390-8621, Japan; 2 Graduate School of Life and Environmental Science, Osaka Prefecture University, 1-1 Gakuen-cho, Nakaku, Sakai, Osaka 599-8531, Japan; 3 The institute of tropical agriculture, Kyushu University, Hakozaki 6-10-1, Higashi-ku, Fukuoka, Fukuoka 812-8581, Japan; 4 Institute of Mountain Science, Shinshu University, Asahi 3-1-1, Matsumoto, Nagano 390-8621, Japan; 5 Department of Mountain and Environmental Science, Interdisciplinary Graduate School of Science and Technology, Shinshu University, 8304 Minamiminowa, Kamiina, Nagano 399-4598, Japan; 6 Brain Science Institute, Tamagawa University, Tamagawagakuen 6-1-1, Machida, Tokyo 194-8610, Japan; 7 Faculty of Agriculture, Ibaraki University, 3-21-1 Chuo, Ami, Ibaraki 300-0393, Japan.

## Abstract

Large blue butterflies, *Phengaris (Maculinea*), are an important focus of endangered-species conservation in Eurasia. Later-instar *Phengaris* caterpillars live in *Myrmica* ant nests and exploit the ant colony’s resources, and they are specialized to specific host-ant species. For example, local extinction of *P. arion* in the U. K. is thought to have been due to the replacement of its host-ant species with a less-suitable congener, as a result of changes in habitat. In Japan, *Myrmica kotokui* hosts *P. teleius* and *P. arionides* caterpillars. We recently showed, however, that the morphological species *M. kotokui* actually comprises four genetic clades. Therefore, to determine to which group of ants the hosts of these two Japanese *Phengaris* species belong, we used mitochondrial *COI*-barcoding of *M. kotokui* specimens from colonies in the habitats of *P. teleius* and *P. arionides* to identify the ant clade actually parasitized by the caterpillars of each species. We found that these two butterfly species parasitize different ant clades within *M. kotokui.*

Several orders of animals are found in ant nests. Some of them depend in some way on ants during their life cycle, which are known as myrmecophiles^1^,^2^. In lepidopteran insects, more than half of Lycaenidae species have associations with ants that range from facultative association to obligate nest parasitism^3^. In order to communicate ants, lycaenid caterpillars and pupae have some myrmecophilous organs, such as dorsal nectary organs, pore cupola organs and tentacle organs, producing nectars and other substances, and organs for sound production^3^. By using these myrmecophilous organs, lycaenids emit chemical and acoustic cues to manipulate their host ants.

Large blue *Phengaris (Maculinea*) butterflies (Lepidoptera: Lycaenidae) are widely distributed in Europe and Asia, and all known species (about 10) are considered to be obligately myrmecophilous. *Phengaris* butterflies are the best-known example of parasitic myrmecophily, and they exhibit a high degree of host-ant specificity^4^. Early instar caterpillars feed on specific host plants (flowers of Lamiaceae, Gentianaceae, or Rosaceae). When they reach the fourth instar, they drop from their host plant to the ground and gain entry to a nest of *Myrmica* ants (Myrmicinae) by using chemical mimicry to cause themselves to be recognized as ant larvae by worker ants, who then carry them into their nest^4^,^5^,^6^. Ant nests are strongly protected by their ant inhabitants. Therefore, if an organism can enter a nest without being attacked by the ants, the nest becomes a safe shelter against natural enemies^1^.

Once they gain entry into an ant nest, the caterpillars grow by exploiting the resources of the ant colony (Fig. 1). *Phengaris* uses two parasitic strategies: “predatory” caterpillars prey on the ant brood, and “cuckoo” caterpillars are fed by the ants via regurgitation^7^,^8^. *Phengaris teleius* and *P. arionides*, which are widely distributed in East Asia, including Japan, are predatory species. Caterpillars following both strategies gain more than 98% of their biomass in the ant nest; thus, these butterfly species are obligate parasites^9^. By the time the fourth-instar caterpillars pupate, the host-ant colonies have suffered serious damage, yet the ants transport these parasites into their nest in their own mandibles.

Because of the high specificity of parasitic *Phengaris* butterflies toward their host plants and ants, both must occur together for a habitat to be suitable for the butterflies. As a result, these butterflies are vulnerable to environmental change, and all species of *Phengaris* are endangered worldwide^10^. Two *Phengaris* species, *P. teleius* and *P. arionides,* are distributed in the Japanese archipelago, and geographic sub-species of *P. teleius* are classified as “Near Threatened” or “Critically Endangered”, and *P. arionides* is classified as “Near Threatened” in the 4th (latest) version of the Japanese red lists^11^. In addition, in March, 2016, the government of Japan’s Ministry of Environment added a sub-species *P. teleius kazamoto* living in Chubu area of central Honshu to the list of “National Endangered Species”, and prohibited the collecting and transferring of it.

To develop a conservation strategy for endangered *Phengari*s butterflies, it is essential to identify their host-ant species. In the United Kingdom, *P. arion* became extinct after its host-ant species was replaced by unsuitable congeners^5^,^12^. Before the 1980s, it was thought that *Phengaris* caterpillars could parasitize any *Myrmica* ant species^13^, but in a comprehensive investigation of host specificity among eight *Myrmica* species and five *Phengaris* species, Thomas, *et al*.^5^ found a one-to-one association between each ant and butterfly species. For example, they found that the survival rate of *P. arion* caterpillars in nests of *M. sabuleti* and *M. scabrinodis* was on average 15% and 2%, respectively^5^. Thus, the major host-ant species of *P. arion* is *M. sabuleti*, and it is difficult for the caterpillars to mature in a nest of *M. scabrinois.*

In past morphological studies, the host-ant species of Japanese *Phengaris* species was identified as *Myrmica kotokui*^2^,^14^,^15^,^16^. However, Ueda, *et al*.^17^ showed that the species recognized as *M. kotokui* on the basis of morphology actually consisted of four genetic clades. Therefore, the host-ant specificity of *Phengaris* needs to be determined not just at the species level but also at the genetic level. Moreover, Ueda, *et al*.^18^ showed that each cryptic clade prefers a different habitat and nesting microhabitat. Thus, *P. teleius*, which inhabits grasslands, and *P. arionides*, which lives in woodlands, might parasitize different ant clades within *M. kotokui*. To determine the true host ant of *P. teleius* and *P. arionides*, we (1) investigated *M. kotokui* colonies in the habitats of *P. teleius* and *P. arionides*, (2) used DNA barcoding to estimate the frequencies of the different ant clades in each habitat, and (3) then identified the ant clade that the caterpillars of each butterfly species actually parasitized.

## Results

The DNA clade of each of the 99 ant colonies collected from the six *Phengaris* habitats was identified by neighbor-joining (NJ) analysis of 470-bp sequences of the mitochondrial *COI* gene (Fig. S1). We found that four belonged to the L1 clade, 67 to the L2 clade, and 28 to the L3 clade (Table 1). Thus, L2 was the dominant clade in the *P. teleius* grassland habitats (86.2–100%), and in the woodland *P. arionides* habitat, all ant colonies belonged to the L3 clade (Table 1). These habitat preferences of the ant clades are congruent with the findings of Ueda, *et al*.^18^.

Next we identified the DNA clade of each ant colony parasitized by *Phengaris* caterpillars. The four ant colonies parasitized by *P. teleius* belonged to L2, and the three ant colonies parasitized by *P. arionides* belonged to L3 (Table 1). Although the sample size is too small for statistical testing, based on the habitat preferences of ants and the parasitic frequency of *Phengaris* caterpillars, we tentatively conclude that *P. teleius* parasitizes L2 colonies and *P. arionides* parasitizes L3 colonies under natural conditions. To determine the specificity of the Japanese *Phengaris—Myrmica* interaction more definitively, additional sampling is essential. During this study, however, we decided not to collect more specimens because we judged that additional collections risked excessively depleting the populations of both butterflies and ants.

## Discussion

We showed that two Japanese *Phengaris* butterfly species apparently parasitize the nests of different ant clades within the *M. kotokui* morphological species. This finding raises the question, does this apparent specificity represent an adaptation on the part of the butterfly, or did the dominance of L2 and L3 clades in grasslands and woodlands, respectively, lead to this apparent one-to-one correspondence without adaptation? It is possible that Japanese *Phengaris—Myrmica* interactions are an example of parasitic adaptation, because preliminary tests indicate that *P. teleius* caterpillars and their host ants have some cuticular hydrocarbons (CHCs) in common (R. Seki, personal communication). To confirm that the adaptation has occurred, in addition to a CHC analysis, acoustic measurements should also be performed to compare the sounds produced by caterpillars and butterfly pupae to those of worker and queen ants, because both chemical and acoustical mimicry by *P. rebeli* caterpillars of their host *Myrmica* ants have been demonstrated^6^,^19^,^20^,^21^,^22^.

In this study, we found cryptic host-ant specificity in *Phengaris* butterflies for the first time. Therefore, to preserve these East Asian butterflies, it is important to maintain their particular host ant clades. Do declines in the number of host-ant colonies in appropriate butterfly habitats drive the extinction of the butterflies? We investigated the ant species composition in the area of the most endangered population of *P. teleius* in habitat E (Table 1). We found that because the soil had acidified, become drier and swampy meadows, suitable micro-habitat to L2 clade, decreased (Table 1), probably as a result of changing agricultural practices, only *Lasius japonicus* (Formicidae) and *Myrmica jessensis,* neither of which are suitable host ants, occurred beneath or near *Sanguisorba officinalis* (Rosaceae), the host plant of early-stage *P. teleius* caterpillars. Because all previous studies showed that *P. teleius* and *P. arionides* in Japan parasitize the nest of *M. kotokui*, we determined that *M. jessensis* may not be a suitable host ant species. There were some reports that the caterpillars of the Japanese *Phengaris* species parasitize the nest of *Aphaenogaster japonica*^14^,^23^, but the ant’s name was mistake for *M. kotokui*^15^. In this area, we were able to fine only two ant nests of the L1 clade, and it was located at the edge of a forest and far from any suitable host plants for the caterpillars. Given the suitable host ant of *P. teleius* is L2 clade, the caterpillars cannot live in any of the ant nests in habitat E. The displacement of ant species in *Phengaris* habitats in the UK has been shown to lead to the extinction of native populations of *P. arion*^5^,^12^. Thomas *et al*.^11^ showed that this high specificity triggered the local extinction of *P. arion. Myrmica scabrinodis* prefers to nest in tall grass, whereas *M. sabuleti* prefers areas where grass height is kept low by herbivory. When herbivores were excluded from the *P. arion* habitat and the grass became high, *M. sabuleti* replaced *M. scabrinodis* and, as a result, the *P. arion* population declined sharply^12^,^24^. On the basis of this finding, in UK sanctuaries for *P. arion*, a suitable environment for the host ant was produced by controlled burning and grazing. Then, once *M. sabuleti* was re-established in the restoration sites, the butterflies were successfully re-introduced from Sweden^5^,^12^. This finding suggests the displacement of ant species in Japan could cause *P. teleius* and *P. arionides* to become extinct. To save Japanese *Phengaris* butterflies from extinction, *Phengaris* populations and their habitats should be surveyed, interactions between the butterflies and ants should be investigated, and the anthropogenic impact on their habitats and hosts should be evaluated.

## Methods

### Parasitization rates of Japanese *Phengaris* on *Myrmica kotokui*

It is not necessary to acquire government permission to collect the ant samples in the concerned regions. However, we got approval to collect the samples from the managers of each butterfly sanctuary. We searched for *M. kotokui* nests in four *P. teleius* habitats (A–E) separated from one another by more than 100 km, and in one *P. arionides* habitat (F). To protect the butterflies, we do not show the detailed collecting site locations here, but each habitat area has a large population of butterflies except for habitat E. The population size of the butterfly may relate to colony density of *M. kotokui* (Table 1). The butterfly population in the habitat (E) with the lowest colony density was much smaller than the others. And the colony density of the ants may relate to soil moisture (Table 1). In each *P. teleius* habitat A–E, *M. kotokui* nested in the muddy soil of a moist grassland, and the swampy meadows decrease may lead to decreasing the colony density of the ants (Table 1). To determine whether *P. teleius* caterpillars were present in a nest, we removed all soil to a depth of 0.5 m within a radius of 1.0 m of the nest entrance. We left all ants, including queen ants, in the colony, except for some worker specimens removed for DNA analysis. In all, we investigated 29 ant colonies in habitat A, 14 in habitat B, 18 in habitat C, 11 in habitat D and 2 in habitat E (Table 1); we found caterpillars in three habitat A colonies and one caterpillar in a habitat B colony, but no caterpillars in habitats C and D (Table 1). Thus, the parasitization rate of *P. teleius* in ant nests was 5.4%, which is lower than rates reported this species in Poland and France^5^,^25^. The lower parasitizing rate in Japan may indicate that the *P. teleius* population is small, or it may be an underestimate, because in our survey we did not completely excavate the ant colonies.

In *P. arionides* habitat F, *M. kotokui* nested in decayed logs in a forest. We opened decayed wood from around each nest to determine whether *P. arionides* caterpillars were present in the nest. We investigated 25 ant colonies in habitat E, and found caterpillars in three of them (Table 1). Thus, the parasitization rate of *P. arionides* in the ant nests was 12.0%. This rate is higher than the *P. teleius* rate (5.4%), but we cannot compare it with rates in other regions because, to our knowledge, this is the first report of the parasitization rate of *P. arionides* in ant nests.

### DNA barcoding of ants

During the nest survey, we collected 10 to 20 worker ants from each colony for DNA barcoding and preserved them in 99.5% EtOH until the analysis. We deposited voucher specimens at the Faculty of Science, Shinshu University, Matsumoto, Japan. We extracted DNA from the whole body of each ant using a DNeasy Blood & Tissue Kit (Qiagen, Hilden, Germany) following the manufacturer’s protocols. Then we amplified the mitochondrial *COI* gene by polymerase chain reaction (PCR) using Takara Ex Taq polymerase (Takara Bio, Shiga, Japan), and the primers MyrCOI-F1 (5′-TA GGR TCR CCT GAT ATA GC-3′) and MyrCOI-R1 (5′-CC AGG TAY YAT TAA AAT ATA AAC TTC-3′)^18^. The reaction was carried out for 30 cycles of 95 °C for 30 s, 50 °C for 30 s, and 72 °C for 40 s. After amplification, the PCR products were purified with ExoSap-IT reagent (USB, Cleveland, Ohio, USA). Both strands were sequenced with a BigDye Terminator v1.1 Cycle Sequencing Kit (ABI, Weiterstadt, Germany) on an ABI 3130 Genetic Analyzer.

The mitochondrial *COI* sequences were edited and aligned with SeqScape v. 2.5 software (ABI, Weiterstadt, Germany). We imported the obtained *COI* dataset into the *COI* dataset of Ueda *et al*.^17^, and then determined the clade of the ants in each colony by a neighbor-joining NJ analysis, performed with MEGA6 software^26^. Although Ueda *et al*.^17^ used both *COI* and *LwRh* sequences to infer the molecular phylogeny, in this study we analyzed only the *COI* sequences because the mutation rate of the *LwRh* gene is slow and it is possible to determine the clade by using only the *COI* gene data. The GenBank accession numbers of the *COI* gene sequences are listed in Table S1.

## Additional Information

**How to cite this article:** Ueda, S. *et al*. Host-ant specificity of endangered large blue butterflies (*Phengaris* spp., Lepidoptera: Lycaenidae) in Japan. *Sci. Rep.* **6**, 36364; doi: 10.1038/srep36364 (2016).

**Publisher’s note**: Springer Nature remains neutral with regard to jurisdictional claims in published maps and institutional affiliations.

## Supplementary Material

Supplementary Figure S1

Supplementary Table S1

## Figures and Tables

**Figure 1:**
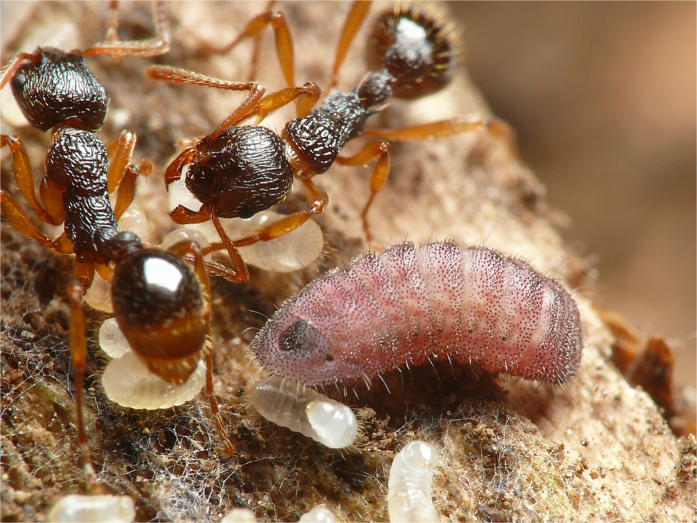
A *Phengaris arionides* caterpillar feeding on larvae belonging to the L3 clade of *Myrmica kotokui* (Photo by T. Komatsu).

**Table 1:** Occurrence of ant clades within *Myrmica kotokui* in six Japanese *Phengaris* habitats (A–F) and the clades of the ant colonies actually parasitized by caterpillars.

*Phengaris* species	Region	Location	Soil moisture	Colony density of *M. kotokui*	No. of ant nests examined	Ant clades of nests			No. of nests with *Phengaris*	Clades of parasitized ants		
						L1	L2	L3		L1	L2	L3
*P. teleius*	Hokkaido	A	++	+++	29	1 (3.4%)	25 (86.2%)	3 (10.3%)	3	0	3	0
	Tohoku	B	+++	+++	14	0 (0%)	14 (100%)	0 (0%)	1	0	1	0
	Tohoku	C	+++	+++	18	0 (0%)	18 (100%)	0 (0%)	0	0	0	0
	Chubu	D	++	++	11	1 (2.8%)	10 (93.1%)	0 (0%)	0	0	0	0
	Chubu	E	+	+	2	2 (0%)	0 (0%)	0 (0%)	0	0	0	0
*P. arionides*	Chu-bu	F	++	+++	25	0 (0%)	0 (0%)	25 (100%)	3	0	0	3

Ant clades were identified by mitochondrial COI barcoding.
