# Corrigendum: Host-ant specificity of endangered large blue butterflies (*Phengaris* spp., Lepidoptera: Lycaenidae) in Japan

**DOI:** 10.1038/srep45613

**Published:** 2017-03-30

**Authors:** Shouhei Ueda, Takashi Komatsu, Takao Itino, Ryusuke Arai, Hironori Sakamoto

Scientific Reports 6: Article number: srep3636410.1038/srep36364; published online: 11 03 2016; updated: 03 30 2017

This Article contains typographical errors in Table 1. For Location E, the ‘L1’ value “2 (0%)” should read “2 (100%)”. Additionally, the Region for ‘*P. arionides*’, “Chu-bu” should read “Chubu”.

