# Supplementary material for: Host-ant specificity of endangered large blue butterflies (Phengaris spp., Lepidoptera: Lycaenidae) in Japan
Source: Sci Rep. 2016 Nov 3;6:36364. doi: 10.1038/srep36364 (PMC5093462; doi:10.1038/srep36364)

Title
Host-ant specificity of endangered large blue butterflies (*Phengaris* spp., Lepidoptera: Lycaenidae) in Japan

Authors
Shouhei UEDA[1,2], Takashi KOMATSU[3], Takao ITINO[2,4], Ryusuke ARAI[5], Hironori SAKAMOTO[6]

Affiliation
[1]Graduate School of Life and Environmental Science, Osaka Prefecture University, 1-1 Gakuen-cho, Nakaku, Sakai, Osaka 599-8531, Japan
[2]Department of Biology, Faculty of Science, Shinshu University, Asahi 3-1-1, Matsumoto, Nagano 390-8621, Japan
[3]The institute of tropical agriculture, Kyushu University, Hakozaki 6-10-1, Higashi-ku, Fukuoka, Fukuoka 812-8581, Japan
[4]Institute of Mountain Science, Shinshu University, Asahi 3-1-1, Matsumoto, Nagano 390-8621, Japan
[5]Department of Mountain and Environmental Science, Interdisciplinary Graduate School of Science and Technology, Shinshu University, 8304 Minamiminowa, Kamiina, Nagano 399-4598, Japan
[6]Brain Science Institute, Tamagawa University, Tamagawagakuen 6-1-1, Machida, Tokyo 194-8610, Japan

Corresponding author
Shouhei Ueda
Graduate School of Life and Environmental Science, Osaka Prefecture University, 1-1 Gakuen-cho, Nakaku, Sakai, Osaka 599-8531, Japan
Tel: +81-72-254-9413
Fax: +81-72-254-9694
E-mail: sueda@envi.osakafu-u.ac.jp

Figure S1 legend
Fig. S1. Neighbor-joining phylogeny of *Myrmica kotokui* estimated by using the 508 bp mitochondrial DNA sequences of cytochrome oxidase I. The numbers next to the branches are the NJ bootstrap support (> 60%).

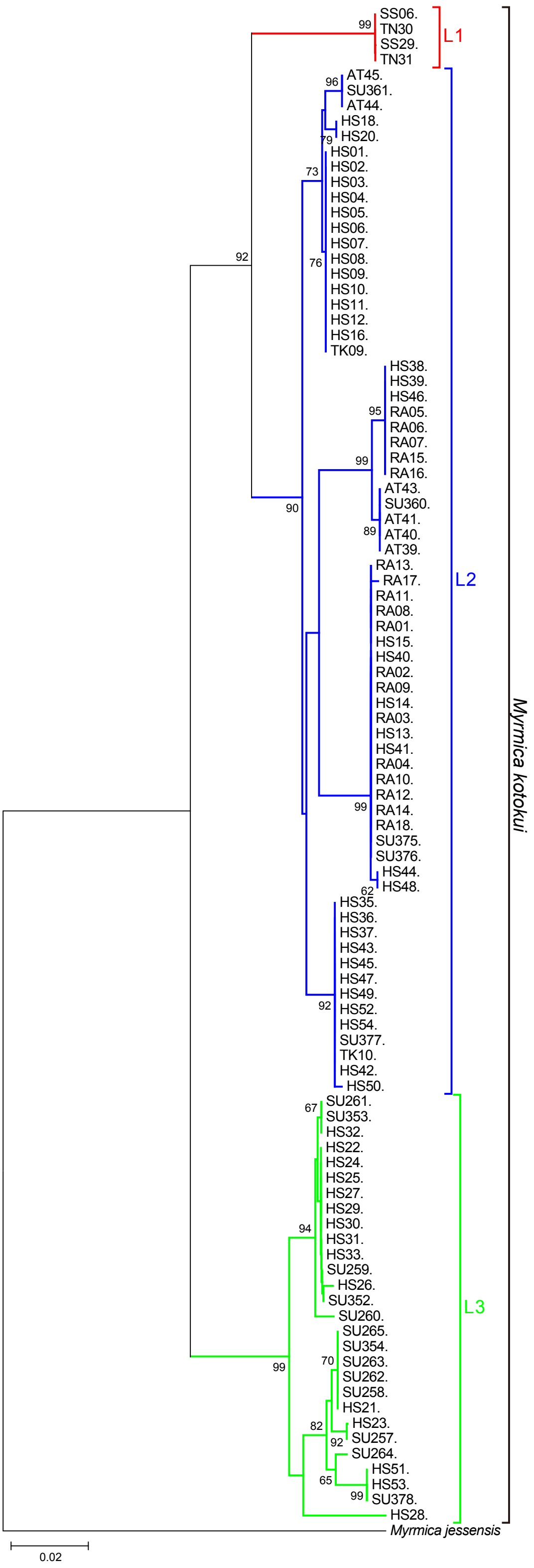

Supplement: Supplementary Figure S1 [file srep36364-s1.pdf]
